# Incidental findings of uncertain significance: To know or not to know - that is not the question

**DOI:** 10.1186/s12910-016-0096-2

**Published:** 2016-02-13

**Authors:** Bjørn Hofmann

**Affiliations:** Norwegian University of Science and Technology, Gjøvik, Norway; Centre for Medical Ethics, University of Oslo, PO Box 1130, Blindern, N-0318 Oslo, Norway

**Keywords:** Incidental findings, Genetic testing, Genome, Exome, Uncertainty

## Abstract

**Background:**

Although the “right not to know” is well established in international regulations, it has been heavily debated. Ubiquitous results from extended exome and genome analysis have challenged the right not to know. American College of Medical Genetics and Genomics (ACMG) Recommendations urge to inform about incidental findings that pretend to be accurate and actionable. However, ample clinical cases raise the question whether these criteria are met. Many incidental findings are of uncertain significance (IFUS). The eager to feedback information appears to enter the field of IFUS and thereby threaten the right not to know. This makes it imperative to investigate the arguments for and against a right not to know for IFUS.

**Discussion:**

This article investigates how the various arguments for and against a right not to know hold for IFUS. The main investigated arguments are: hypothetical utilitarianism, the right-based argument, the feasibility argument, the value of knowledge argument, the argument from lost significance, the empirical argument, the duty to disclose argument, the avoiding harm argument; the argument from principle, from autonomy, from privacy, as well as the argument from the right to an open future. The analysis shows that both sides in the debate have exaggerated the importance of incidental findings.

**Summary:**

Opponents of a right not to know have exaggerated the importance of IFUS, while proponents have exaggerated the need to be protected from something that is not knowledge. Hence, to know or not to know is not the question. The question is whether we should be able to stay ignorant of incidental findings of uncertain significance, if we want to. The answer is yes: As long as the information is not accurate and/or actionable: ignorance is bliss. When answering questions that are not asked, we need to think twice.

“Medical science has made such tremendous progress that there is hardly a healthy human left.”

Aldous Huxley

## Background

“Do not answer questions not asked.” This general proverb has found many expressions, e.g., in “The Knight’s Tale” from 2001 William exclaims: “Herald, do not answer questions you do not know the answer to!” and in *Men in Black* Agent K states: “Don’t ask questions you don’t want to know the answer to.” While such statements go well in fiction, in science things are different. Research oftentimes gives us new knowledge that we did not ask for, but that changes the world. In predictive medicine, and in genetics in particular, this is different too. New knowledge is found – knowledge that is important and needs to be communicated. However, when giving answers to questions people do not have, the answers have to be reliable and helpful. That is, they have to be accurate and actionable. If not, people are generally considered to have a right not to be informed. This is highly topical as new omics, such as integrative Personal Omics Profile (iPOP), generates a wealth of data (>3 billion measurements), finding a huge amount of variants (>3 million single nucleotide variants) with uncertain or unknown significance [1]. Even for well characterized genes, such as BRCA1 and BRCA2, there are variants of uncertain significance [2].

The right not to know is recognized in international and national regulation. According to the European Convention on Human Rights and Biomedicine Article 10.2 “[e]veryone is entitled to know any information collected about his or her health. However, the wishes of individuals not to be so informed shall be observed”. In the World Medical Association 1981/1995 Article 7d it is stated that “the patient has the right not to be informed on his/her explicit request, unless required for the protection of another person’s life,” and the UNESCO Declaration on the Human Genome, Article 5c claims that “[t]he right of every individual to decide whether or not to be informed of the results of genetic examination and the resulting consequences should be respected”.

This “right not to know” has been heavily debated [3–16]. In particular, it has been argued that technological progress in genomics (and other –omics) makes the right not to know obsolete [17–23]. The ACMG recommendations for reporting of incidental findings in clinical exome and genome sequencing lists 57 (56) genes and 24 disorders that should be tested when using exome or genome sequencing as these are associated with phenotypes for which “preventive measures and/or treatments [are] available and disorders in which individuals with pathogenic mutations might be asymptomatic for long periods of time.” [24]. Therefore, they favor ignoring patients’ preferences and recognize that in doing so they violate patient autonomy and a right not to know [24]. Moreover, genetics professionals appear to think that to know is better than not to know [25] even when the information is actionable [26].

Although these recommendations have stirred a lively debate [27–46] there has been little attention to what kind of information is gained from such tests in practice and how that influences the right not to know. Is the information accurate and actionable, i.e., do they provide helpful knowledge or confusing non-validated information? A recent case referred in The New York Times may illustrate this:

*“Jennifer was 39 and perfectly healthy, but her grandmother had died young from breast cancer, so she decided to be tested for mutations in two genes known to increase risk for the disease. When a genetic counselor offered additional tests for 20 other genes linked to various cancers, Jennifer said yes. The more information, the better, she thought. The results, she said, were “surreal.” She did not have mutations in the breast cancer genes, but did have one linked to a high risk of stomach cancer. In people with a family history of the disease, that mutation is considered so risky that patients who are not even sick are often advised to have their stomachs removed. But no one knows what the finding might mean in someone like Jennifer, whose family has not had the disease”* [47].

The provided information is not helpful and it is fair to ask whether Jennifer has a right to be ignorant. There are divided opinions about this. However, as the authors in NYT point out, what is revealed in Jennifer’s case is not knowledge, but risk information causing questions, uncertainty, and anxiety [47]. Hence, one could argue that the discussion of a right not to know is irrelevant, as what is presented is not knowledge. It is barely information. It is data with unknown significance.

On the other hand it is argued that this is information about a potential life-threatening risk factor and “that clinicians and laboratory personnel have a fiduciary duty to prevent harm by warning patients and their families about certain incidental findings and that this principle supersedes concerns about autonomy” [24]. Let us for the sake of the argument accept that there is significant uncertainty related to incidental findings in exome and genome analysis. Even ACMG recognizes that “there are insufficient data on penetrance and clinical utility to fully support these recommendations” and “that there was insufficient evidence about benefits, risks, and costs of disclosing incidental findings to make evidence-based recommendations” [24]. Moreover, they acknowledge that to a large extent “the Working Group drew upon the clinical judgment of its members” [24].

As it is argued that we have to discard the right not to know in exome and genome analysis, and this appears to extend to incidental findings of uncertain^1^ significance^2^ (IFUS), it becomes imperative to investigate the arguments for and against a right not to know for IFUS. Do the arguments hold? Are they relevant? It will be argued that few of the arguments for and against a right not to know hold for IFUS. Even more, they are not relevant, as IFUS at best is information, and not knowledge^3^. Hence, there is a right to remain ignorant about test results with uncertain significance. Discussing the right not to know in such cases only confuses the matter. Let me review some of the arguments in order to investigate their relevance, starting with the arguments against the right not to know.

## Discussion

### Arguments against a “right not to know”

The ACMG recommendations argue from a kind of *hypothetical utilitarianism*: the information must be conveyed to the person as this *will potentially improve* the person’s health and potentially even *save the life* of the person. The argument is in line with traditional arguments for protecting the best interest of a person [48, 49]. “It seems uncontroversial to state that almost everyone would (and perhaps even should) want to know genetic information that could lead to an intervention that would prevent or mitigate serious morbidity or mortality” [18]. One may, of course, argue that it is quite unclear what “would” and “could” means in such statements, and that they are based on biased conception of beneficence, ignoring risk and harm. Let that be as it may. In the case of IFUS it is fundamentally uncertain whether there is any benefit what so ever. Genetic risk scores appear to have lower discriminative accuracy than clinical risk factors, and common complex disorders are only partially heritable. “Most genetic variants may only alter the disease susceptibility risk by a factor of 1.1–1.6, and usually a large number of genetic variants will have a bearing on the risk … In most cases of polymorphic variants, the predictive value would be too small for any intervention to be appropriate ….” [50]. Hence, when accuracy is poor, if existing at all, and actionability is speculative, the argument does not hold. Moreover, there is little evidence of impact on behavior change [49, 51].

There are also *right-based arguments* against a right not to know. They may be based on third parties rights, e.g. the hypothetical right not to know in the future of one person may be overruled by the right to know (now) of another person, e.g., a parent or guardian: “To mask or withhold the incidental finding is to state that the child’s right not-to-know supersedes the parent’s opportunity to discover a lifethreatening risk factor” [24]. It may also be argued that the right not to know has to be balanced against other rights and other kinds of ethically relevant interests, as it is not a right that outdoes all other considerations [52]. “The idea that there is a general right not to know health information that trumps other interests should be rejected. Such a presumed right must be balanced against other competing rights and other interests.” [53]. E.g., in genetic testing of children it is argued that the benefits of disclosing predictive genetic information to a parent can override the goal of deferring a child’s choice to be tested for that information until adulthood [54].

These arguments presume either that somebody else’s right to know or potential benefit trumps (a potentially acceptable) right not to know. However, in the case of IFUS there is no violation of others’ rights as there is nothing to know (in terms of accuracy and/or actionability), and it is hard to see how rights to know or other interests outrun the right not to know, if there is little to know.

Yet other arguments against a right not to know are *arguments from (re)classification*. They try to undermine a “right not to know” by arguing that it is not a *right* [52], only an interest, and that the interest not to know has to be balanced against other interests [7, 55]. However, even if we accept this argument, proponents would have to argue that the interest of being informed about IFUS overrides an interest not to be informed about IFUS.

A related argument against the right not to know claims that it is *not a fundamental or a basic right*. The right not to know is not found among the basic rights presented in theories of rights. It is derived from other, more fundamental rights, such as freedom or autonomy: “The argument in favour of a right not to know is based on the autonomy of the individual” [8, 56]. Moreover, the right not to know does not always protect the right it is derived from [21, 52, 57]. Again, even if we accept these arguments, it does not follow that there are other more basic rights that trump a right not to be informed about something that is neither accurate nor actionable.

There are also counterarguments from *feasibility*. E.g., for something to be a right it must be possible to exercise it [58, 59]. Or in another phrasing: to know whether people have a preference not to know, you have to inform them [4]. However, the traditional ought-implies-can-argument does not apply in the IFUS case. Here there is no clear imperative to inform, so the argument falls. On the contrary it illustrates shortcomings of the feasibility argument: if there is little to know, it cannot undermine (or support) a right to know it.

A more principled counter-argument is that *knowledge is a good in itself* according to which a“right to remain ignorant” is a contradiction. This argument often refers to Aristotle’s conception of man: “all men by nature desire to know” [52] or a Kantian perspective where the “right to remain in ignorance” is an irrational attitude [13, 60, 61]. It may of course also refer to a Socratic/Platonic conception of knowledge to be action-guiding. Hiding information hampers autonomy, and hence, promotes paternalism. In the case of IFUS, it is hard to understand how information can increase autonomy. It is not irrational to refuse information that is not accurate and/or actionable. On the contrary, to claim that you need to know about IFUS, is paternalistic.

There are also counter-arguments from *lost significance* (or altered relevance). For predictive genetic testing or carrier testing of monogenetic conditions a right not to know makes sense, but in the new setting this right has lost its significance [24, 38]. E.g., ignorance made sense with Huntington’s disease, where little could be done, but it does not make sense in a world of extensive genetic tests of potential great benefit [18, 62–64]. This is a *petitio principii,* as it presumes what has to be proven, i.e., that something can be done. In case of IFUS the information is uncertain and not actionable, and hence, of little relevance.

There are also *empirical counter arguments* (in addition to the non-feasibility argument mentioned above), e.g., that most people would like to be informed [18] and that only few like not to be informed [53]. In addition to the basic “naturalistic fallacy,” reasoning from *is* to *ought*, we do not know what people prefer in the IFUS case. Studies of people’s preferences ask whether they would like to have information that is accurate and actionable, and seldom about uncertain and inactionable information. We do know, however, that genetic professionals tend to think it is good to be informed [25, 26].

Correspondingly, it has been argued that people “are not as autonomous as is generally assumed by the defenders of the right not to know,” [53] and hence, the right not to know has reduced weight. This line of argument refers to studies that show that those few who do not want to know are not fully autonomous [53]. If they were autonomous, they would like to know. This kind of argument raises the obvious question of why we think that only those who do not want to know have reduced autonomy? If people in general have reduced autonomy, the imperative to (be able to) inform them may be as undermined as the right not to know. Be that as it may, in the case of IFUS, the argument fails, as there is nothing to *know*. There seems to be a fair agreement that autonomy requires understanding, and IFUS does not promote understanding of one’s health.

There is also a counterargument from *professional ethics*, as the right not to know goes contrary to the doctor’s “duty to disclose” [4]. However, it is not clear that a duty to disclose includes IFUS. On the contrary, and as the case in the introduction illustrates, informing about IFUS may challenge the principle of *primum non nocere.*

The same goes for another argument from principle, e.g. that a right not to know goes against the *principle of solidarity* or that it can *harm* others [65] as it prohibits disclosing vital information to family members. Again, even if one acknowledges and appreciates the solidarity principle or the harm principle, the argument loses force because the information is not vital in the case of IFUS, as it is not accurate or actionable. It is far from obvious that it avoids harm to others. On the contrary, it may cause psychological harm to the person and family members if disclosed.

The *legal counterargument* does not bite either. One version of the argument goes like this: if a person, who has claimed his right not to know, is given information that was lifesaving, it is hard to see how a jury or judge would blame the persons or institution that gave him the information. Conversely, in a court case with a person who has waived his right not to know would claim that “if I was told that this could be of vital importance to my life and health, I would obviously not have waived the right,” the court may very well agree. Hence, the right not to know would not be valid in judicial practice, and only therefore, at best, be of theoretical interest. However, in the case of IFUS, the lifesaving premise does not hold. If the information is not actionable, it is far from obvious that a court or judge will blame anybody for not informing.

Hence, the arguments against the right not to know do not hold for IFUS. Let me therefore now turn to arguments for a right to know before summarizing the arguments in Table 1.

**Table 1:** Overview of the arguments for and against a *right not to know* and short outline of the problems with these arguments

Con	Pro
Hypothetical utilitarianism: The information will be important in the future. Problem: *petitio principii*	Avoiding harm: Being ignorant to avoid (psychological) harm of information. Problem: Information about IFUS is not knowledge.
Rights-based arguments: Third party rights or interests override the rights of the individual. Problem: No defined third party rights or interests due to lack of accuracy and actionability	Argument for future flourishing (and liberty): Information about IFUS may reduce (future) flourishing and liberty. Problem: IFUS is not knowledge (and should not reduce flourishing)
Arguments from (re)classification: It is not a right, but an interest, or not a basic right. Problem: Not relevant for IFUS	Autonomy based arguments: not knowing is exercising autonomy. Problem: IFUS does not provide knowledge.
It is not feasible: It is not possible to exercise a right not to know. Problem: Irrelevant as there is nothing to know.	Privacy: there is a right to retain a private sphere without intrusion. Problem: It is not clear that providing IFUS data is an intrusion.
Knowledge is a good thing in itself: A right not to know is a contradiction of this good. Problem: There is not knowledge.	Empirical arguments: People with accurate tests for severe diseases prefer not to be informed. Problem: reasoning from IS to OUGHT
Argument from lost significance: A right not to know is not relevant in the age of genomics (with potential great benefit). Problem: petitio principio	Absence of duties: no duty not to inform, i.e., no right not to be informed. Problem: absence of a duty does not correspond with a right.
Empirical argument: Most people want to be informed. Problem: reasoning from IS to OUGHT	The right to an open future: ignorance preserves the potential of open future choices. Problem: IFUS does not represent information that threatens an open future (formally).
Argument from duty to disclose. Problem: counters *primum non nocere,* and that there is no knowledge to disclose	
Argument from principle (of solidarity or) of avoiding harm (to others): not knowing, may harm others. Problem: no knowledge, no harm	
Legal argument: Would not be litigated for giving vital information. Problem: IFUS is not vital information.	

### Arguments for a right not to know

One of the main arguments for a right not to know has a (utilitarian) basis in *avoiding harm*. A person may perfectly well want to stay ignorant in order to avoid psychological harm [4]. In the case of genetic testing of children such harms have been specified: “The potential harms caused by childhood genetic testing might include damage to the child’s self-esteem, distortion of the family’s perceptions of the child, loss of future adult autonomy and confidentiality, discrimination against the child in education, employment or insurance, and adverse effects on the child’s capacity to form future relationships” [66]. If IFUS do not harm a person in these or other ways, this argument may be dismissed. However, if IFUS is able to cause any (psychological) harm, one may argue that the avoiding-harm-argument holds. But not for defending the right not to know, but only for a right not to be informed about IFUS.

Another related argument for the right not to know reasons from *future flourishing (and liberty)*. Knowledge of potential but uncertain risks may reduce human prospering and flourishing. “I should not go hiking, as my genome is not so strong.” I.e., the knowledge may reduce a person’s actual flourishing or liberty. Again, the information of IFUS is not knowledge, and one could argue that it should not be able to reduce anyone’s flourishing or liberty. If it does, one makes erroneous inference from the information. However, if IFUS shows to reduce persons flourishing, one could argue that the argument holds. Again, at best the argument supports a right not to be informed about IFUS.

There are also various *autonomy based arguments* for a right not to know. E.g. it is argued that choosing not to know is a way to exercise one’s autonomy, i.e., a right to informational self-determination [4]. The patient, and not the professional, should decide what information to be conveyed to the patient. It is the patient who takes on the responsibility and the action of deciding which information he or she wants or does not want to receive [49]. Another argument from (lack of increased) autonomy goes like this: when no alternative treatments or actions are available, knowing does not increase autonomy. Hence, you may as well stay ignorant. Not knowing does not make you subject to paternalism (and i.e., not reduced autonomy). On the contrary, conveying information without the request of the person is a type of paternalism [65]. It can also be argued that present ignorance preserves and promotes the potential of future autonomous choices and that it can be defended from “*the right to an open future*” – in particular for children [67, 68].

In the case of IFUS, we do not even need to enter the debate on whether you can be autonomous if you do not want to know [49, 54, 68, 69]. It does not seem to threaten a person’s present or future autonomy to refuse information about test results that are not accurate or actionable. However, this does not provide a knock down argument for the right not to know. It only provides an argument for a right not to be informed about IFUS.

There are also arguments for the right not to know from the basic *right to privacy*. Laurie argues that “[c]ontrol of information about ourselves must be an essential part of any concept of ourselves as autonomous persons, but “control” should not be limited merely to control of who has access to that information. It should also include the facility not to accept the information *ab initio*. A concept of “control” which is wide enough to encompass this notion permits us to retain a private sphere that is truly our own.” [7]. Although this may justify the non-generation of knowledge in the first place, it is more challenging to use this to argue for a right not to know existing vital information about oneself. However, in the case of IFUS, this is straight forward, as neither the generation, nor the delivery of information, is of known vital importance. However, it may be argued that IFUS does not represent an intrusion into the private sphere, as the meaning of IFUS is unclear. But, as the example with Jennifer in the introduction illustrates, the information may definitely matter. Although, this is not an argument for a right not to know in general, it may be used to argue for not being informed about IFUS.

As with the counter-arguments to the right not to know, there are also *empirical arguments* for such a right. Many reasonable and autonomous adults choose not to know genetic information of outmost importance to their health. Moreover, some do insist in not knowing such information. E.g., up to 80 % of adults with a family history of Huntington’s disease refuse genetic testing [70–72]. As people who can take a very accurate test for a severe disease,^4^ do not want to know, then others should be given the same option for IFUS. However, this argument reasons from IS to OUGHT. In addition, the empirical premise for the argument is not true in the case of IFUS, as there is little evidence about whether people do or do not want to be informed about IFUS.

There is also an argument for the right not to know from the *absence of duties*. If there is no duty to inform persons about health risks, e.g., about IFUS on an individual level, then there is a right not to know. However, the absence of a duty does not necessarily correspond to a right, so this argument has to be rephrased: If there is a duty not to inform persons about health risks that are not of vital importance for their lives, then there is a corresponding right not to know health related information that is not of vital importance to one’s life. So far, there are few knock down arguments for a duty not to inform persons about IFUS. It may be reasonable not to inform, but there is no imperative.

Hence, there are a series of arguments for the right not to know, and many problems with them. Table 1 summarizes the arguments for and against the right not to know that have been reviewed so far.

Hence, both the arguments against and for a right not to know do not apply to the case of IFUS. As shown, there are many reasons for this, but one common underlying factor is that IFUS does not represent knowledge. Data - yes, information – maybe, but knowledge – no. Hence, much of the debate on incidental findings of uncertain significance is misplaced. “Looking at the entire genome will reveal “incidental” findings, which are unrelated to the clinical request, as well as a number of genetic variants for which the meaning remains unclear” [49]. Both sides of the debate appear to have hyped the importance of such findings. Those who want to inform have exaggerated the importance of the findings for saving lives, while those who want to stay ignorant have exaggerated the need to be protected from something that is not knowledge (information without significance).

### Limitations and reflections

This study is not exhaustive. There may of course be other arguments for and against a right not to know than those presented here. However, would they make a difference? Most arguments for or against refer to some content of the knowledge involved, e.g., either that it is accurate or actionable.

Accordingly, one could dismiss the whole issue of whether there is a right to know or not to know for IFUS in the first place. We could have concluded directly that to know or not to know, that is not the question. The question is: can I trust the information and will it make any change? However, there are three reasons why this investigation has been important. First, the right not to know, and the duty to inform, have been at the core of ethical debates on incidental findings including IFUS. Second, tests that are considered to render significant information, are in practice IFUS. Third, the arguments for or against a right not to know may have been valid for a right not to be informed about IFUS. Actually, several arguments support a right not to be informed about IFUS: if information from genetic tests is not accurate or actionable, I may well be entitled to stay uninformed about these from several perspectives: autonomy, privacy, harm, open future, and flourishing/liberty. However, if well informed about the limited value of IFUS, it is not clear that the information harms or reduces autonomy, future flourishing or liberty. Empirical studies are needed to clarify this and are most welcome.

The interesting thing with using IFUS as a case to discuss the right not to know is that it unpeels a series of arguments that presuppose significant outcomes. E.g., as series of empirical arguments become invalid, as they presuppose that the information “will prevent serious disease and perhaps even save the life” [18] or that most people want information of incidental findings: “…. while the right not to know may seem appropriate in the abstract, people’s views change once they are presented with a case where a specific piece of lifesaving information is available.” [18]. All such arguments presuppose that the information is of importance. However, with IFUS it is not.

In the same way as many of the arguments for informing people of incidental findings presuppose that the information is accurate and actionable, I have here presupposed that it is not, or more precisely, I have defined that IFUS is not accurate or actionable. Hence, I have tried to twist the presuppositions of the con-arguments. It is an empirical question how many test results that are IFUS, and it is an interpretative issue when they are IFUS. But if IFUS exist, I have tried to show that most of the arguments for and against a right to be informed do not apply.

It is of course interesting from a principled point of view to discuss whether there is a right not to know (or a duty to inform). However, when such principled debates, using idealized conditions of accurate and actionable information, are used to regulate practice where such conditions are not met, it is appropriate to revisit the debate and the arguments with real world conditions, e.g., like those of Jennifer at the outset of this article.

It is also an open question what an accurate test result means. For various types of diagnostic tests there are standards of accuracy, e.g., in terms of sensitivity and specificity. Incidental findings for genetic tests can be considered to parallel screening, as there is no indication for the test or the pre-test probability is low. For screening tests there are specific requirements, usually towards 99 % sensitivity and specificity. Accuracy can also be assessed in terms of predictive values (negative and positive predictive values). Genetic test results tend to have low predictive values [50]. Nevertheless, it is a normative question where we set the limits.

It is also an open question what we consider as actionable. I have here considered actions improving a person’s health, because I consider those to be most important in a health care setting. Actions with other purposes may of course be of interest.

In the area of co-production of knowledge one should be careful to give fixed definitions of knowledge. However, the point here has not been to enter the diverse and inconclusive debates on epistemology, but to display that there is a difference between data, information, and knowledge. The point is that dumping data or inaccurate information on people is not making them knowledgeable.

Although I do support a right not to be informed about IFUS, and this article has identified flaws with arguments for and against the right not to know, it is inconclusive with respect to this right in general. Nevertheless, I have revealed a significant asymmetry in how the opponents and proponents appreciate the uncertainties of potential benefits and the uncertainties of potential harms. The point here is that specific uncertainties (in accuracy and actionability) undermine the potentiality of both benefits and harms. Hyping benefits or harms does not help people like Jennifer^5^.

## Conclusion

Most arguments for and against a right not to know fail for IFUS. Mainly because they contain hyped premises of incidental findings’ vital significance or their potential harm respectively. Hence, to know or not to know, that is not the question. The question is: can I trust the test results and will they make any difference? I.e., will I become diseased and can anything be done? IFUS cannot answer these questions. Accordingly, if I cannot trust a test result, and/or (if the result is accurate but) nothing can be done to improve my health, there appear not to be any compelling reason that I should be informed, if I do not want to be. Correspondingly, one could claim a right not to be given inaccurate and/or inactionable information. In the case of IFUS ignorance is bliss. When answering questions that are not asked, we need to think twice.

## Footnotes

1 In the literature there are many terms for findings of uncertain significance: they are “unspecified,““unknown,” “unclear”. They are also frequently coined “variants of uncertain significance” (VUS), where “uncertain” can be replaced with the terms above. Uncertainty here means that one may know of potential outcomes, but that the probability is unknown. Hence, IFUS is not risk information, as risk contains information of both outcomes and probabilities.

2 Significance here is defined as meaning that a test result is accurate (so it can be trusted) and/or that it is actionable, i.e., that something can be done with the effect of improving a person’s (future) health. Technically accuracy is given in terms of sensitivity and specificity, and it does not mean that these have to be 100 %. Moreover, significance does not include that something may be done in the future, e.g., due to new treatments in the future. The “or” between “accurate” and “actionable” acknowledges accurate knowledge may be of value to a person even if it is not actionable.

3 Accordingly, knowledge here means that information about a potential health condition following from a test result is accurate or actionable.

4 A positive test result is partly (“indirectly”) actionable in terms of family planning (including PGD).

5 It may of course be argued that the finding in Jennifer’s case was not an “incidental” finding, but something that was proposed to better assess her (breast) cancer risk. It may have been discussed in a pre-test consultation highlighting the fact the interpretation of positive results in any of 20 the other genes will turn out to be (very) difficult and/or limited. We do not know Jennifer’s reflection when she assented, nor how she interpreted the pre-test information. We only know that she defines the results “surreal,” indicating that the result came as a surprise. I am most thankful to one of the reviewers for pointing this out.

## References

[1] Chen R, Mias GI, Li-Pook-Than J (2012). Personal omics profiling reveals dynamic molecular and medical phenotypes. Cell.

[2] Cheon JY, Mozersky J, Cook-Deegan R (2014). Variants of uncertain significance in BRCA: a harbinger of ethical and policy issues to come?. Genome Med.

[3] Uninformed consent. New tests raise questions over right not to know test results. Hospital ethics / American Hospital Association. 1995.11(4):10–1. 10144095

[4] Andorno R (2004). The right not to know: an autonomy based approach. J Med Ethics.

[5] Green J, Kentish J (1995). The right not to know HIV-test results. Lancet.

[6] Kielstein R, Sass HM (1992). Right not to know or duty to know? Prenatal screening for polycystic renal disease. J Med Philos.

[7] Laurie GT (1999). In defence of ignorance: genetic information and the right not to know. Eur J Health Law.

[8] Malpas P (2005). The right to remain in ignorance about genetic information--can such a right be defended in the name of autonomy?. N Z Med J.

[9] Neumann H (1980). Patients have the right not to know. Med World News.

[10] Raikka J (1998). Freedom and a right (not) to know. Bioethics.

[11] Sears RA (1965). The right not to know. S D J Med.

[12] Shaw MW (1987). Testing for the Huntington gene: a right to know, a right not to know, or a duty to know. Am J Med Genet.

[13] Takala T (1999). The right to genetic ignorance confirmed. Bioethics.

[14] Weaver KD (1997). Genetic screening and the right not to know. Issues Law Med.

[15] Wilson J (2005). To know or not to know? Genetic ignorance, autonomy and paternalism. Bioethics.

[16] Herring J, Foster C (2012). “Please don’t tell me”. The right not to know. Camb Q Healthc Ethics.

[17] Austad T (1996). The right not to know--worthy of preservation any longer? An ethical perspective. Clin Genet.

[18] Berkman BE, Hull SC (2014). The “right not to know” in the genomic era: time to break from tradition?. Am J Bioeth.

[19] Bortolotti L, Widdows H (2011). The right not to know: the case of psychiatric disorders. J Med Ethics.

[20] Pennings G (2001). The right to privacy and access to information about one’s genetic origins. Med Law.

[21] Rhodes R (2006). Genetic testing: is there a right not to know?. MCN Am J Matern Child Nurs.

[22] Van Leeuwen E, Hertogh C (1992). The right to genetic information: some reflections on Dutch developments. J Med Philos.

[23] Zinberg RE (2006). Genetic testing: is there a right not to know?. MCN Am J Matern Child Nurs.

[24] Green RC, Berg JS, Grody WW (2013). ACMG recommendations for reporting of incidental findings in clinical exome and genome sequencing. Genet Med.

[25] Lemke AA, Bick D, Dimmock D (2013). Perspectives of clinical genetics professionals toward genome sequencing and incidental findings: a survey study. Clin Genet.

[26] Yu J-H, Harrell Tanya M, Jamal Seema M (2014). Attitudes of Genetics Professionals Toward the Return of Incidental Results from Exome and Whole-Genome Sequencing. Am J Hum Genet.

[27] Anastasova V, Blasimme A, Julia S (2013). Genomic incidental findings: reducing the burden to be fair. Am J Bioeth.

[28] Biesecker LG (2013). The Nirvana fallacy and the return of results. Am J Bioeth.

[29] Borgelt E, Anderson JA, Illes J (2013). Managing incidental findings: lessons from neuroimaging. Am J Bioeth.

[30] Christenhusz GM, Devriendt K, Dierickx K (2013). Disclosing incidental findings in genetics contexts: a review of the empirical ethical research. Eur J Med Genet.

[31] Christenhusz GM, Devriendt K, Van Esch H, et al. Ethical signposts for clinical geneticists in secondary variant and incidental finding disclosure discussions. Medicine, health care, and philosophy. 2014. doi: 10.1007/s11019-014-9611-8. [published Online First: Epub Date]| 10.1007/s11019-014-9611-8 25407129

[32] Clayton EW, Haga S, Kuszler P (2013). Managing incidental genomic findings: legal obligations of clinicians. Genet Med.

[33] Costain G, Bassett AS (2013). Incomplete knowledge of the clinical context as a barrier to interpreting incidental genetic research findings. Am J Bioeth.

[34] Dal-Re R, Katsanis N, Katsanis S (2014). Managing incidental genomic findings in clinical trials: fulfillment of the principle of justice. PLoS Med.

[35] Eckstein L, Garrett JR, Berkman BE (2014). A framework for analyzing the ethics of disclosing genetic research findings. J Law Med Ethics.

[36] Garrett JR (2013). Reframing the ethical debate regarding incidental findings in genetic research. Am J Bioeth.

[37] Gliwa C, Berkman BE (2013). Do researchers have an obligation to actively look for genetic incidental findings?. Am J Bioeth.

[38] Greenbaum D (2014). If you don’t know where you are going, you might wind up someplace else: incidental findings in recreational personal genomics. Am J Bioeth.

[39] Jarvik GP, Amendola LM, Berg JS (2014). Return of genomic results to research participants: the floor, the ceiling, and the choices in between. Am J Hum Genet.

[40] Kleiderman E, Knoppers BM, Fernandez CV (2014). Returning incidental findings from genetic research to children: views of parents of children affected by rare diseases. J Med Ethics.

[41] Klitzman R, Appelbaum PS, Chung W (2013). Return of secondary genomic findings vs patient autonomy: implications for medical care. Jama.

[42] Klitzman R, Buquez B, Appelbaum PS (2014). Processes and factors involved in decisions regarding return of incidental genomic findings in research. Genet Med.

[43] Parens E, Appelbaum P, Chung W (2013). Incidental findings in the era of whole genome sequencing?. Hast Cent Rep.

[44] Rehder CW, David KL, Hirsch B (2013). American College of Medical Genetics and Genomics: standards and guidelines for documenting suspected consanguinity as an incidental finding of genomic testing. Genet Med.

[45] Ulrich M (2013). The duty to rescue in genomic research. Am J Bioeth.

[46] Vayena E, Tasioulas J (2013). Genetic incidental findings: autonomy regained?. Genet Med.

[47] Grady D, Pollack A. Finding Risks, Not Answers, in Expanding Array of Gene Tests. In: The New York Times. New York edn. New York: The New York Times; 2014: A17.

[48] Borry P, Howard HC, Senecal K (2009). Direct-to-consumer genome scanning services. Also for children?. Nat Rev Genet.

[49] Borry P, Shabani M, Howard HC (2014). Is there a right time to know? the right Not to know and genetic testing in children. J Law Med Ethics.

[50] Janssens AC, van Duijn CM (2008). Genome-based prediction of common diseases: advances and prospects. Hum Mol Genet.

[51] Marteau TM, French DP, Griffin SJ, et al. Effects of communicating DNA-based disease risk estimates on risk-reducing behaviours. The Cochrane database of systematic reviews. 2010(10): Cd007275. doi: 10.1002/14651858.CD007275.pub2. [published Online First: Epub Date]. 10.1002/14651858.CD007275.pub2 PMC12853416 20927756

[52] Harris J, Keywood K (2001). Ignorance, information and autonomy. Theor Med Bioeth.

[53] Helgesson G, Eriksson S, Swartling U (2007). Limited relevance of the right not to know--reflections on a screening study. Account Res.

[54] Abdul-Karim R, Berkman BE, Wendler D (2013). Disclosure of incidental findings from next-generation sequencing in pediatric genomic research. Pediatrics.

[55] Laurie GT (2004). A response to Andorno. J Med Ethics.

[56] The Human Genetics Commission (2000). Whose hands on your genes?.

[57] Eriksson S (2004). Should results from genetic research be returned to research subjects and their biological relatives?. TRAMES – A Journal of the Humanities and Social Sciences.

[58] Laurie GT (2001). Challenging medical-legal norms. The role of autonomy, confidentiality, and privacy in protecting individual and familial group rights in genetic information. J Legal Med.

[59] Bottis MC (2000). Comment on a view favoring ignorance of genetic information: confidentiality, autonomy, beneficence and the right not to know. Eur J Health Law.

[60] Rhodes R (2000). Autonomy, respect, and genetic information policy: a reply to Tuija Takala and Matti Hayry. J Med Philos.

[61] Hayry M, Takala T (2001). Genetic information, rights, and autonomy. Theor Med Bioeth.

[62] de Wert G (1992). Predictive testing for Huntington disease and the right not to know. Some ethical reflections. Birth Defects Orig Artic Ser.

[63] Erez A, Plunkett K, Sutton VR (2010). The right to ignore genetic status of late onset genetic disease in the genomic era; Prenatal testing for Huntington disease as a paradigm. Am J Med Genet A.

[64] Hayden MR (2003). Predictive testing for Huntington’s disease: a universal model?. Lancet Neurol.

[65] Takala T (2001). Genetic ignorance and reasonable paternalism. Theor Med Bioeth.

[66] Duncan RE, Delatycki MB (2006). Predictive genetic testing in young people for adult-onset conditions: where is the empirical evidence?. Clin Genet 2006.

[67] Feinberg J, Aiken W, LaFollette H (1980). The child’s right to an open future. Whose child? parental rights, parental authority and state power.

[68] Davis DS (1997). Genetic dilemmas and the child’s right to an open future. Hast Cent Rep.

[69] Ross KM, Reiff M (2013). A perspective from clinical providers and patients: researchers’ duty to actively look for genetic incidental findings. Am J Bioeth.

[70] Creighton S, Almqvist EW, MacGregor D (2003). Predictive, pre-natal and diagnostic genetic testing for Huntington’s disease: the experience in Canada from 1987 to 2000. Clin Genet.

[71] Robins Wahlin TB (2007). To know or not to know: a review of behaviour and suicidal ideation in preclinical Huntington’s disease. Patient Educ Couns.

[72] Tassicker RJ, Teltscher B, Trembath MK (2009). Problems assessing uptake of Huntington disease predictive testing and a proposed solution. Eur J Hum Genet.

