# Erratum to: Incidental findings of uncertain significance: To know or not to know – that is not the question

**DOI:** 10.1186/s12910-016-0119-z

**Published:** 2016-06-23

**Authors:** Bjørn Hofmann

**Affiliations:** Norwegian University of Science and Technology, Gjøvik, Norway; Centre for Medical Ethics, University of Oslo, PO Box 1130, Blindern, N-0318 Oslo, Norway

## Erratum

Following publication of the original article in BMC Medical Ethics [1], it was brought to my attention that the following sentences are incorrect:

“Moreover, genetics professionals appear to think that to know is better than not to know [25] even when the information is actionable [26].” This sentence should read: Moreover, genetics professionals appear to think that to know is better than not to know [25] even when the information is not actionable [26].

“Let me therefore now turn to arguments for a right to know before summarizing the arguments in Table 1.”

This sentence should read: Let me therefore now turn to arguments for a right not to know before summarizing the arguments in Table 1.

I apologise for the inconvenience this may have caused.
